# Effect of Nebulized Colistin on the Ventilator Circuit: a Prospective Pilot Case- Control Study from a Single Cancer Center

**DOI:** 10.4084/MJHID.2015.032

**Published:** 2015-04-20

**Authors:** Iyad M Ghonimat, Lama H Nazer, Flsteen Aqel, Mohammad K Mohammad, Feras I Hawari, Jennifer Le

**Affiliations:** 1 Respiratory Therapy Services, King Hussein Cancer Center, Amman, Jordan; 2 Department of Pharmacy, King Hussein Cancer Center, Amman, Jordan; 3 ACDIMA Arab Company for Drug Industries & Medical Appliances, Amman, Jordan; 4 Pulmonary and critical care, King Hussein Cancer Center, Amman, Jordan; 5 University of California, San Diego Skaggs School of Pharmacy and Pharmaceutical Sciences, San Diego, CA, USA.

## Abstract

Nebulized colistin (NC) is used for the treatment of pneumonia due to multidrug- resistant Gram-negative bacteria. In this one-year case-control study, our objective was to evaluate the effect of NC on the ventilator circuit (VC) components. The case group consisted of 25 mechanically-ventilated patients who received NC for the treatment of nosocomial pneumonia while the control group was 25 mechanically-ventilated patients who did not receive NC. Respiratory therapists inspected the VC every 4 hrs and whenever a ventilator alarm was reported. The VC component was changed if the alarm did not subside after necessary measures were performed. Patients from both groups were treated at the adult medical/surgical intensive care unit at King Hussein Cancer Center. In the case group, 22 (88%) patients required changing at least one of the circuit components (flow sensor, exhalation membrane, or nebulizer kit). The median number of changes (range) per patient of the flow sensor, exhalation membrane, and nebulizer kit were: 2 (1–3), 2 (1–6), and 1 (1–2), respectively. Large amounts of white crystals, which resembled the colistin powder, were reported on the replaced VC components. The flow sensor was changed in 2 control patients, but white crystals were absent. Crystals obtained from one case subject were confirmed to be colistin by chromatographic mass spectroscopy. Further studies are needed to evaluate the effect of crystal formation on the efficacy of NC and clinical outcomes.

## Introduction

Nebulized colistin (NC) has been widely used over the last decade in critically ill patients for the treatment of multidrug resistant (MDR) Gram-negative bacteria, mainly *Pseudomonas aeruginosa* and *Acinetobacter baumannii*.^1^ Several studies suggested that nebulized colistin, as adjunct to systemic antibiotic treatment, improves bacteriological and clinical response in patients with nosocomial pneumonia.^2^–^6^ In addition, the American Thoracic Society-Infectious Disease Society of America Ventilator Associated Pneumonia guidelines recommended adjunctive aerosolized antibiotics in patients with MDR infections who are not responding well to therapy.^7^

Despite its increased use, the safety data of NC remains limited. In general, aerosolized anti-infectives are attractive owing to their relative lack of systemic toxicities.^8^ However, serious adverse effects have been reported with NC, including bronchospasm requiring mechanical ventilation and leading to death.^8^ Recently, we raised the concern of crystal formation and frequent changes of the ventilator circuit components in mechanically ventilated patients treated with NC.^9^ Up to our knowledge, there have been no previous reports of these observations. To explore this further, we conducted this prospective pilot case-control study to evaluate the effect of NC on the components of the mechanical ventilator in critically ill patients with nosocomial pneumonia.

## Patients and Methods

### Study site and subjects

The study was conducted in a 12-bed adult medical/surgical intensive care unit (ICU) of a 170-bed comprehensive teaching cancer center, King Hussein Cancer Center, in Amman, Jordan. The ICU manages oncology-related and non-oncology related critical illnesses in cancer patients. The ICU has a closed-unit model with high intensity staffing, in accordance with the Leapfrog standards. This model was implemented several years ago and has demonstrated improved clinical outcomes in critically ill oncology patients.^10^

The study included 25 consecutive mechanically- ventilated patients who received NC for the treatment of nosocomial pneumonia due to multidrug resistant gram negative bacteria (case group). The typically administered dose of NC was 1 million units every 8 hrs. The control group consisted of 25 consecutive mechanically ventilated patients who did not receive any nebulized medications while they were on mechanical ventilation. Patients on mechanical ventilation or NC for <24 hrs were excluded.

### Study design and methods

This investigation was a one-year prospective pilot case-control study (February, 2012 to April, 2013), approved by the local institutional review board (IRB). The respiratory therapists on a daily basis inspected the mechanical ventilator circuits for all patients every 4 hrs and whenever a ventilator alarm was reported. The ventilator used was the Galileo Gold Ventilator (Hamilton Medical AG, Via Nova, Switzerland), along with the Intersurgical Flextube™ circle breathing system and the Hamilton single-use flow sensor. A ventilator circuit component was changed if the alarm did not subside, after all, necessary measures were performed and proved ineffective.

The NC was prepared at the pharmacy using the product Colomycin® (Forest Laboratories, UK), which is approved for inhalation and intravenous uses. The preparation of NC was done under aseptic conditions, according to the manufacturer’s recommendations: one million units of colistimethate sodium was dissolved in 2 ml of normal saline, and then refrigerated, with an expiration of 24 hours from the time of preparation. The NC was administered by the respiratory therapist who was assigned to the ICU patients. All respiratory therapists working in the ICU are trained in managing critically ill patients and in the administration of aerosolized medications. The NC was given after the administration of any nebulized or inhaled bronchodilator using the nebulizer kit (Plasti-Med®), and delivered over 20 to 30 minutes. The nebulizer kit allowed the administration of the aerosolized colistin, with a particle size of 2.7 micron MMAD (mass median aerodynamic diameter), without interrupting the ventilation cycle and according to the mechanical ventilator pressure. During the administration of NC, the respiratory therapist remained in proximity to the patient. Once the administration of NC was completed, the nebulizer kit was disconnected by the respiratory therapist, cleaned thoroughly, and stored by the patient’s bedside to use for subsequent doses.

The patient demographics, length of stay, and ICU mortality were recorded. In addition, the type of ventilator component changed (flow sensor, exhalation membrane, or nebulizer kit), the day(s) on which the ventilator component was changed and, the number of changes was noted. When changing any of the ventilator components, the respiratory therapist documented the reason for change and described any unusual findings. A ventilator check list was used when inspecting the ventilator circuit and to record the findings. Subjects were monitored until they were extubated or transferred out of the ICU, which ever occurred first.

To identify the content of the white crystals, they were investigated in residue samples by the Arab Company for Drug Industries & Medical Appliances (ACDIMA) BioCenter, which is an international research organization specialized in clinical studies and bioanalysis. An exhalation membrane that was changed and had significant amount of white crystals was placed in a tightly-closed container and then transported immediately with ice to the analytical site of the ACDIMA BioCenter. The sample was stored under -80°C until the time of testing. We used the Colomycin® (Forest Laboratories, UK) product as a control in the analysis. The identity of the residue sample was investigated using Liquid chromatography coupled with an electrospray ionization tandem mass spectrometry platform (Triple Quad Tandem Mass Spectrometer, API 4000 LC-MS/MS). The collected crystals were dissolved in sufficient quantity of methanol and injected into the instrument. MS/MS spectra from samples, the accumulated residue, and the colistin drug powder were scanned, and their fragmentation patterns were elucidated under the conditions listed in Table 1.

### Statistical analysis

Descriptive statistics were used to report the results. Continuous data were reported using mean with standard deviation (SD) and/or median with range while categorical data were reported as counts and percentages. The Chi-square or Fisher Exact test was used to compare the categorical data while the t- test or Kruskal-Wallis test was used to compare the continuous data. A significance criterion of p<0.05 was used in the analysis. All analysis was performed using SAS version 9.1 (SAS Institute Inc, Cary, NC).

## Results

The demographics and outcomes of the control and case groups are outlined in Table 2. Both groups were similar, except for a longer duration of ICU stay and a longer duration of mechanical ventilation in the case group, compared to the control group. During mechanical ventilation, both groups received adaptive support ventilation mode, with a minute ventilation (MV) ranging from 100–150%, PEEP between 5–14 cm H_2_O, and fraction of inspired oxygen (FiO_2_) between 40–100%.

**Figure 1:**
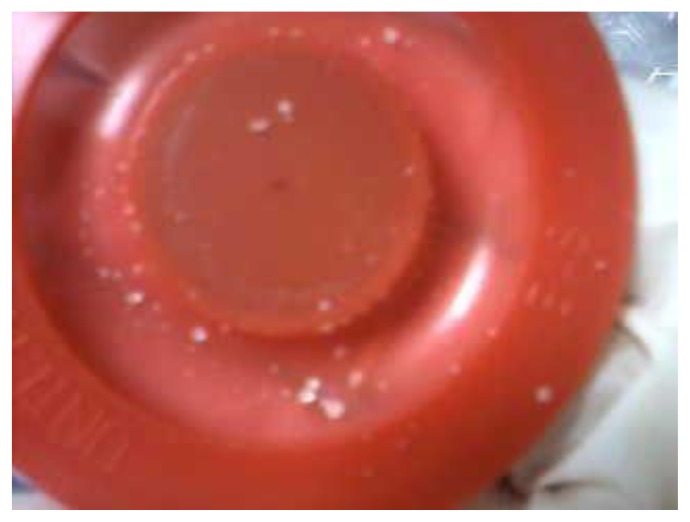
Exhalation membrane; white crystals noted in the center.

**Figure 2:**
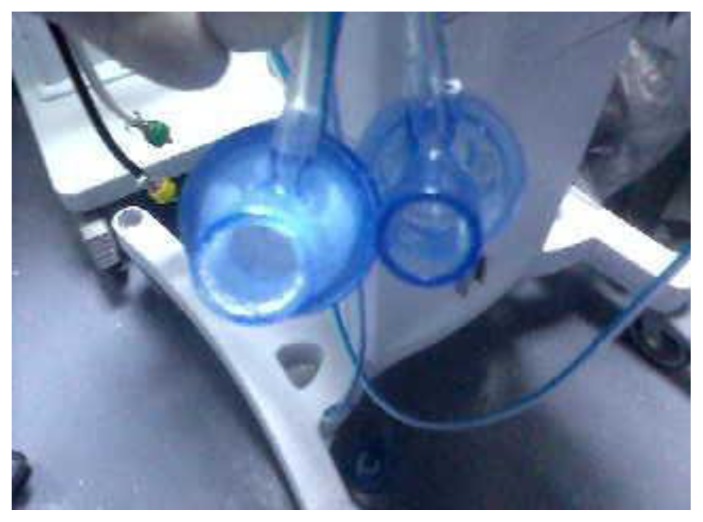
Flow sensors; white crystals noted in the internal part for a patient who received nebulized colistin (left), while the other flow sensor for a patient who did not receive nebulized colistin (right)

During mechanical ventilation, all subjects in both groups received albuterol, administered by a metered dose inhaler. Subjects did not receive any aerosolized antibiotics, except for the use of NC by the case group. All patients in the case group received intravenous and nebulized colistin for the treatment of nosocomial pneumonia, including ventilator-associated pneumonia, due to multidrug resistant *Acinetobacter baumannii*. Among those who received NC, 22 (88%) subjects required changing at least one of the circuit components (flow sensor, membrane, or nebulizer kit). At a median duration of NC use of 10 days, the median number of changes (range) per person in the flow sensor, exhalation membrane, and nebulizer kit were: 2 (1–3), 2 (1–6), and 1 (1–2), respectively (Table 3). Formation of large amounts of white crystals, which resembled the colistin powder, was observed in all ventilator components that were changed (Figure 1 and 2). The median duration to crystal formation was four days, (range 2–5), and crystals were found primarily in the exhalation membrane. All subjects with NC required at least one exhalation membrane change at a median of 7 days (range, 2–25) after initiating NC; this was followed by changing the flow sensor and nebulizer kit in 82% and 41% of the cases, respectively.

**Figure 3:**
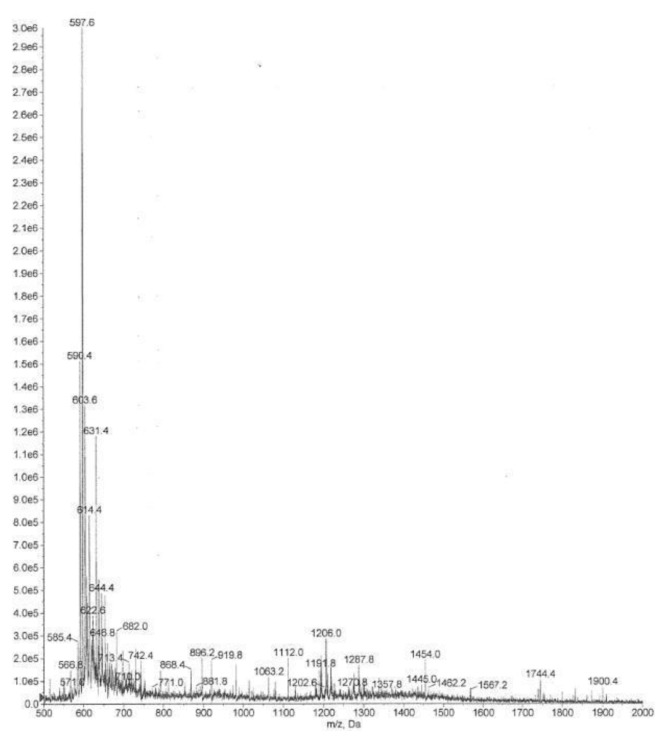
MS/MS spectra for the colistin product (Colomycin®)

**Figure 4:**
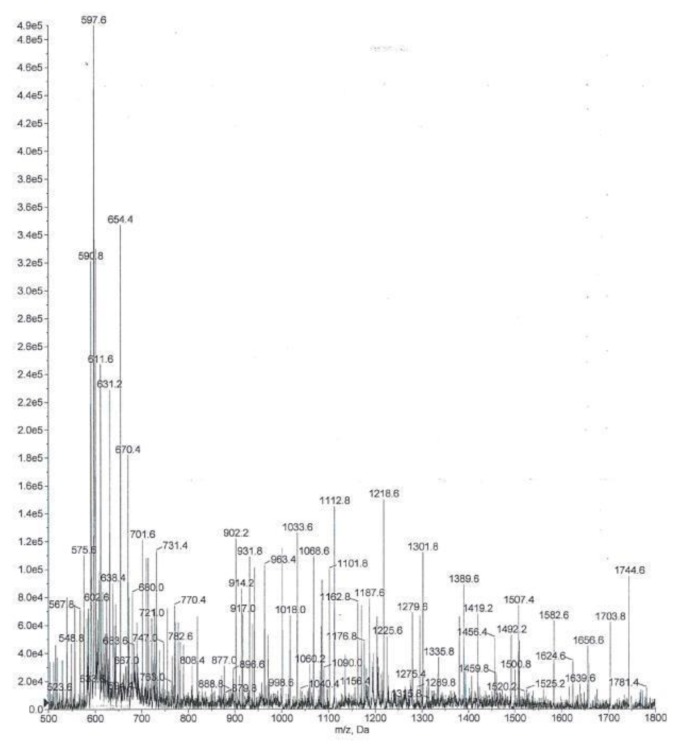
MS/MS spectra for colistin drug powder sample isolated from the ventilator.

In the control group, the flow sensor was changed for two patients (8%). There were no white crystals noted on the flow sensor, and the replacement appeared to be due to a defect in the flow sensors. The results of the MS/MS detection and identification of crystals obtained from one subject using NC demonstrated the same fragmentation pattern, as that obtained from the Colomycin® and both were consistent with polymyxin behavior (Figures 3 and 4). Polymyxins have a complex fragmentation behavior, and the spectra reflects a similar pattern for both samples with base- peak ion mass at 597.56 (for the collected residue sample) and at 597.61 (for Colomycin®).

## Discussion

This study demonstrated the high incidence of crystal formation on various parts of the ventilator circuit in patients who received NC. The crystals were found as early as two days after initiating NC. In our previous case reports, crystal formation and the change in the ventilator component were observed as early as one day after initiating NC.^9^ The aftermath of crystal formation was the requirement for change in at least one of the ventilator components in the majority of subjects receiving NC.

Based on the chromatography analysis completed for one subject, it was confirmed that the crystals were comprised of colistin. Although we did not analyze the crystals for all subjects, we assumed that the content of the crystals were the same since they all had similar general appearances. The origination of the crystals is unknown. We hypothesize that colistimethate sodium starts to crystallize shortly after its preparation, and once administered, the high flow induced by the ventilator stimulates the formation of more crystals that are apparent in the ventilator circuit. The crystals formed in the prepared product were most likely non- visible because the respiratory therapist typically inspects the nebulizer solution prior to administration.

The observations reported in this study raise two major issues: First is regarding the amount of colistin reaching the site of infection in the lungs. The concern of sub-therapeutic doses reaching the site of infection might be considered, and future studies should focus on measuring the amount of colistin that is lost in the ventilator circuit and through exhalation to determine the actual amount of colistin reaching the lungs. The second issue is whether the crystal formation has an effect on the patients' ventilation and/or the ability of the ventilator machine to operate accurately. The flow sensor in the ventilator circuit provides the clinician with valuable data about the patients' ventilation measures so that the ventilator setting can be adjusted accordingly. However, if there are crystals formation in the flow sensor, this might interfere with the accuracy of provided data.

Based on the results of this study, we started changing the flow sensor every three days for all mechanically ventilated patients receiving NC. This is significantly more than what we typically do for our ventilated patients who are not receiving NC and also more than the manufacturer's recommendations. In fact, the manufacturer recommends changing the flow sensor every two weeks, and we typically change it every 1–2 weeks. However, with our concern about the possible effect of the crystals on the functionality of the flow sensor, and since the ventilator alarm is a sign of the late stage of crystal formation, we decided to change the flow sensor more frequently.

This study had some limitations. First it was conducted in a single center therefore affecting its reproducibility in another setting. Secondly, we did not analyze the content of all crystals nor did we quantify the amount of colistin that had crystallized. In addition, we did not match the subjects in the case and control groups, but when comparing the two groups, they were similar in demographics. Finally, we did not correlate the findings with clinical outcomes.

## Conclusions

This is the first study to describe a major complication associated with the administration of NC. The use of NC was associated with crystal formation and subsequent changes of the ventilator circuit components in about 90% of the subjects. Further investigations are imperative to confirm our findings and to evaluate the clinical implications of this complication.

## Figures and Tables

**Table 1:** Conditions used in the drug analysis.

Probe type:	Turbo V spray	Polarity:	+ve
Scan Type:	MRM	Curtain Gas (CUR):	10
Ion Source:	ESI	Ion Source Gas 2(GS2):	20
Ion Source Gas 1(GS1);	20	Temperature (TEM):	600
Ion Spray Voltages(IS):	5500	Collision Gas (CAD):	10
Interface Heater (IHE):	ON	Entrance Potential (EP):	10
Declustering Potential(DP):	120	Collision Cell Exit potential (CXP):	15
Collision Energy (CE):	35	Curtain Gas (CUR):	23

**Table 2:** Demographics and clinical characteristics.

Characteristic	Patients who received nebulized colistin *(n=25)*	Patients who didn’t receive nebulized colistin *(n=25)*	P-value
Sex, male, n (%)	17 (68)	15 (60)	0.370
Age (year):			
median (range)	58 (19–82)	51 (50–77)	0.372
APACHE II score			
median (range)	23 (9–35)	24 (9–38)	1.00
ICU length of stay:			
median (range), days	18 (4–61)	9 (1–59)	0.028
Died in the ICU, n (%)	8 (72)	19 (76)	0.747
Cancer Type, n (%)			
Hematologic	5 (20)	2 (8)	0.747
Solid tumor	20 (80)	23 (92)	
Duration of mechanical ventilation:			
median (range), days	17 (2–45)	7 (1–35)	0.0018
Duration of nebulized colistin:			
median (range), days	10 (1–20)	N/A	N/A
Other aerosolized medications, n (%)			
Albuterol inhaler	25 (100)	25 (100)	N/A

**Table 3:** Subjects with crystal formation and obstruction of the ventilator circuit during nebulized colistin treatment (n=22)

Characteristic	
First report of crystal formation	
median (range), days	4 (2–5)
Location of first crystals identified	
Membrane, n (%)	20 (91)
Flow sensor, n (%)	1 (4.5)
Nebulizer kit, n (%)	1 (4.5)
Flow sensor change	
Subjects that required flow sensor change, n (%)	18 (82)
Number of times changed per person, median (range)	2 (1–3)
Day of change, median (range)	9 (3–25)
Membrane change:	
Subjects that required membrane change, n (%)	22 (100)
Number of times changed per person, median (range)	2 (1–6)
Day of change, median (range)	7 (2–25)
Nebulizer kit change:	
Subjects that required nebulizer kit change, n (%)	9 (40.9)
Number of times changed, median (range)	1 (1–2)
Day of change, median (range)	9 (3–25)
